# Effect of Grain Size on Carburization Characteristics of the High-Entropy Equiatomic CoCrFeMnNi Alloy

**DOI:** 10.3390/ma14237199

**Published:** 2021-11-25

**Authors:** Hyunbin Nam, Jeongwon Kim, Namkyu Kim, Sangwoo Song, Youngsang Na, Jun-Ho Kim, Namhyun Kang

**Affiliations:** 1 Department of Joining Technology, Korea Institute of Materials Science, Changwon 51508, Korea; hbnam12@kims.re.kr (H.N.); swsong@kims.re.kr (S.S.); 2 Production Team, Gestamp Kartek Co., Ltd., Gimhae 50875, Korea; jeokim@kr.gestamp.com; 3 Department of Authorized Nuclear Inspection, Korea Institute of Materials Science, Changwon 51508, Korea; nkkim@kims.re.kr; 4 Department of Special Alloy, Korea Institute of Materials Science, Changwon 51508, Korea; nys1664@kims.re.kr; 5 Dongnam Regional Division, Korea Institute of Industry Technology, Yangsan 50635, Korea; 6 Department of Materials Science and Engineering, Pusan National University, Busan 46241, Korea

**Keywords:** high-entropy alloys, carburization, grain size, carbide precipitates, microstructure, hardness distribution

## Abstract

In this study, the carburization characteristics of cast and cold-rolled CoCrFeMnNi high-entropy alloys (HEAs) with various grain sizes were investigated. All specimens were prepared by vacuum carburization at 940 °C for 8 h. The carburized/diffused layer was mainly composed of face-centered cubic structures and Cr_7_C_3_ carbide precipitates. The carburized/diffused layer of the cold-rolled specimen with a fine grain size (~1 μm) was thicker (~400 μm) than that of the carburized cast specimen (~200 μm) with a coarse grain size (~1.1 mm). In all specimens, the carbides were formed primarily through grain boundaries, and their distribution varied with the grain sizes of the specimens. However, the carbide precipitates of the cast specimen were formed primarily at the grain boundaries and were unequally distributed in the specific grains. Owing to the non-uniform formation of carbides in the carburized cast specimen, the areas in the diffused layer exhibited various carbide densities and hardness distributions. Therefore, to improve the carburization efficiency of equiatomic CoCrFeMnNi HEAs, it is necessary to refine the grain sizes.

## 1. Introduction

High-entropy alloys (HEAs) are in the limelight as new materials that can substitute functional steels for specific applications [1,2]. HEAs, specifically CoCrFeMnNi, are known to have excellent mechanical properties at room and cryogenic temperatures. They stabilize solid solution phases owing to their high mixing entropies and avoid the formation of embrittling intermetallic compounds [3,4,5,6]. However, the CoCrFeMnNi HEA has a moderate surface hardness and yield strength because no phase transformation and second phase are formed during solidification and production [7]. Therefore, the application of such HEAs is limited in fields where surface performance is required, such as wear and fatigue resistance. Thus, surface processing is indispensable for expanding the application range of HEAs.

To improve the surface performance of HEAs, surface modifications, such as cladding [8,9] and plasma spraying [10,11,12], have been investigated. Recently, studies on the carburization of CoCrFeNi HEAs have been reported [7,13]. Carburization is a process based on the diffusion of carbon atoms into the metal matrix to form a carburized layer [14,15]. Peng et al. [7] studied the surface hardening of CoCrFeNi HEA by gaseous carburization at 470 °C for 40 h. The carbide-free supersaturated interstitial solid solution of carbon in the face-centered cubic (FCC) formed on the surface of the HEA enhances the surface hardness of the HEA owing to the solid solution strengthening caused by interstitially dissolved carbon atoms. Zhang et al. [13] studied the surface modification of equiatomic CoCrFeNi HEA by solid carburization at 920 °C for 10 h. Two types of carbide precipitates (M_7_C_3_ and M_23_C_6_) were formed on the surface, improving the surface hardness and wear resistance of the carburized surface.

Because the HEAs are multi-component alloys composed of five or more main components, the mechanism to produce carbide phases formed through the diffusion of carbon atoms is more complex. The mechanical properties of the base metal (BM) and weld for HEA vary with respect to the grain size [16,17]. As the grain size decreases, the strength normally increases with a decreasing ductility. The cast and rolled HEAs normally have a large and small grain size, respectively. Thus, a study of the carburization characteristics according to the grain sizes of the HEAs is required to improve the surface performances of such materials.

As mentioned earlier, there are few previous studies related to the carburization of the HEA. The carburization is a technology to improve the surface hardness on the HEA and carbon normally diffuses faster intergranularly than intragranularly. Therefore, this study investigates the effect of grain sizes on the carburization properties of CoCrFeMnNi HEAs. To access various grain sizes, we used cast and cold-rolled HEAs with a significant variation in grain sizes. Microstructural characterization, phase identification, and hardness distribution were conducted to understand the carburization mechanism with respect to the grain sizes of the HEAs.

## 2. Materials and Methods

The HEA ingots used in this study were produced by vacuum induction melting. CoCrFeMnNi HEA ingots were prepared by homogenizing the ingot at 1100 °C for 24 h. The defect-free part of the HEA ingot was thinly sliced to prepare the HEA cast plates with a thickness of 1.5 mm. Furthermore, the manufacturing steps of the cold-rolled HEA plates were as follows: Homogenization of the slab at 1100 °C for 24 h; hot rolling at 500 °C from 16 to 2 mm with furnace cooling; cold rolling at the room temperature (25 °C) from 2 to 1.5 mm. The specimens 20 × 20 × 1.5 mm) fabricated from the cast and cold-rolled HEA (Co_0.2_Cr_0.2_Fe_0.2_Mn_0.2_Ni_0.2_) were used to analyze the structural characteristics of the matrix.

After preparing each specimen, vacuum carburization was performed on the equipment with automatically controlled process parameters. To reduce the temperature difference between the surface and center area in each specimen, the temperature was increased gradually for 2 h from 25 to 940 °C (recrystallization start temperature of austenitic stainless steel with the same FCC structure as CoCrFeMnNi HEAs). After that, the soaking temperature was maintained for 10 min. In the carburizing process, a mixture of C_2_H_2_ (400 L) and H_2_ (200 L) was injected with a pressure of 4 mbar, and the carburizing atmosphere was maintained for 1–2 min by a pulse-type process. In the diffusing process, C_2_H_2_ (40 L) and H_2_ (40 L) were injected at a pressure of 1 mbar. A complete cycle consisted of carburization followed by diffusion, and the duration of the diffusion stage was increased from 1 to 60 min for each cycle. After the diffusion process, the carburized specimens were rapidly cooled with nitrogen gas for 30 min to prevent grain coarsening.

The formation of the carburized layer and cross sectional microstructure of the specimens were observed using light optical microscopy (LOM) and backscattered electron (BSE) mode of scanning electron microscopy (SEM). Phase analysis was performed using X-ray diffraction (XRD) to identify the crystal structures of the carburized layer and matrix. XRD was performed at a scan speed of 2°/min, within a range of 20–90°, voltage of 40 kV, and current of 30 mA using Cu Kα radiation. To analyze the component behavior between the carburized layer and matrix, macro-mapping and line analysis were performed using electron probe microanalysis (EPMA). The carburizing behavior of each specimen was confirmed by electron backscattered diffraction (EBSD) with an inverse pole figure (IPF) and a phase map. To observe the hardness distribution between the carburized layer and matrix, the Vickers hardness was measured with a load of 4 gf (39.23 mN) and at a dwell time of 10 s. The hardness was measured at an interval of 0.05 mm from the carburized layer to the matrix.

## 3. Results

### 3.1. Microstructure and Mechanical Properties of the HEA BM

**Figure 1:**
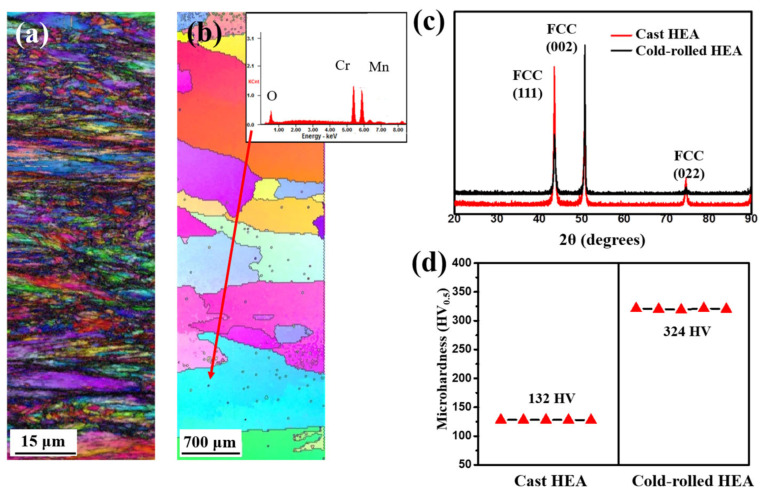
Microstructure and hardness distribution of the various BMs: IPF maps of (**a**) cold-rolled HEA BM and (**b**) cast HEA BM, (**c**) XRD pattern, and (**d**) hardness distribution.

Figure 1a,b shows the representative IPF maps of the cold-rolled and cast HEA BMs, respectively. The microstructure of the cold-rolled HEA BM had fine grains of approximately 1 μm or less during cold rolling (Figure 1a). In the cast HEA BM, coarse grains (approximately 1.1 ± 0.2 mm) were observed after the homogenization treatment (Figure 1b). Furthermore, the black dots detected in Figure 1b corresponded to the CrMn oxides. The grain sizes of the cast and cold-rolled HEA BMs were significantly different. Figure 1c shows the XRD patterns of the cast and cold-rolled BMs. The XRD patterns of each BM indicate a simple FCC solid-solution phase, which is in agreement with previous investigations [18,19,20]. Figure 1d shows the hardness distributions of the cast and cold-rolled HEA BMs. The average hardness values of the cast and cold-rolled HEA BMs were approximately 132 and 324 HV, respectively. The large hardness of the cold-rolled HEA BM was associated with the small grain size and the dislocation accumulated during the cold-rolling process [21,22].

### 3.2. Microstructural Behavior of Carburized/Diffused Layer with Respect to the Grain Sizes of the BMs

**Figure 2:**
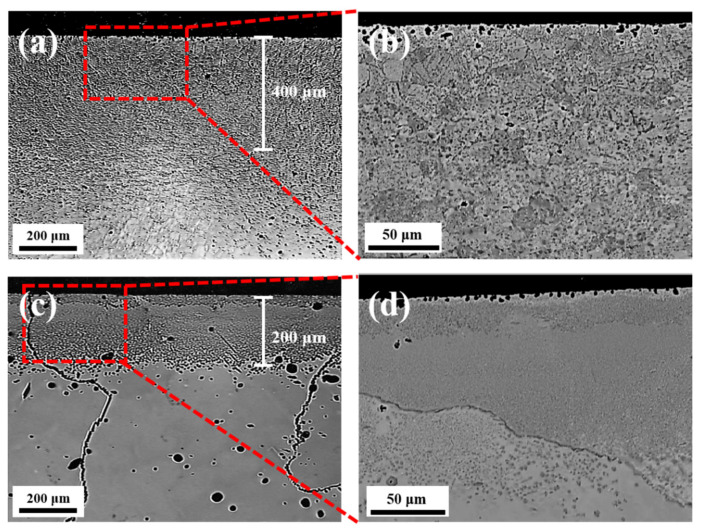
Cross sectional carburization layers: (**a**) LOM and (**b**) SEM-BSE images enlarged for cold-rolled specimen and (**c**) LOM and (**d**) SEM-BSE images enlarged for cast specimen.

Figure 2a,b shows the cross-sectional carburization region of the cold-rolled specimen measured by LOM and SEM, respectively. The dark area on the surface is the region affected by carburization, and the white grain in the center region is the BM of cold-rolled and cast HEAs. The black particles observed on the top surface of both BMs are probably carbon particles, as the BSE mode indicates black as the colour for light elements. It was difficult to differentiate between the carburized surface and BM in the cold-rolled specimen (Figure 2a), probably because the small particles were uniformly distributed in the carburized layer of the cold-rolled specimen (Figure 2b). However, the carburization region and cast BM were clearly identifiable in the cast HEA specimen (Figure 2c), which was probably because of with the non-uniform distribution of tiny particles in each grain (Figure 2d). The thickness of the carburization region for the cast HEA was approximately 200 μm, which is thinner than that of the cold-rolled HEA (~400 μm).

**Figure 3:**
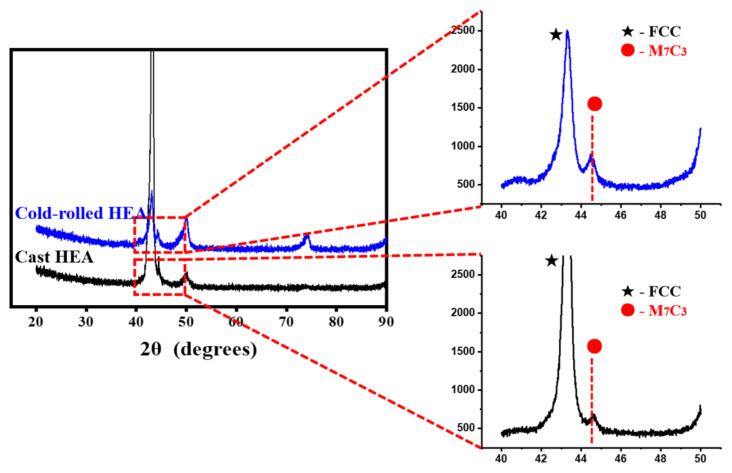
XRD patterns of the carburized layer for the cold-rolled and cast specimens.

Figure 3 shows the crystal structure acquired by XRD for the carburization region of the cold-rolled and cast specimens. The FCC as well as precipitates were observed owing to the carburization process in all specimens. The non-carburized specimens of the cold-rolled and cast HEAs exhibited FCC peaks, as shown in Figure 1c. However, as in Figure 2, a large amount of precipitates and particles were formed on the surface after vacuum carburization in all specimens. The peaks of the precipitates were composed of signals from M_7_C_3_. Zhang et al. [13] reported that HEA-carburized specimen was almost composed of M_7_C_3_ and M_23_C_6_ carbides. However, the M_23_C_6_ peaks were not observed in the XRD results because the M_7_C_3_ carbides occupied most of the surface in the cold-rolled and cast HEA-carburized specimens in this study (Figure 3).

**Figure 4:**
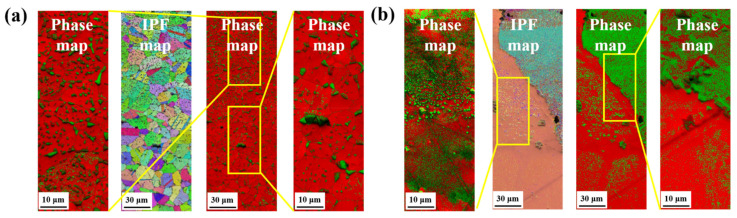
Carbide precipitation behavior of carburized specimens on various BMs: (**a**) Cold-rolled and (**b**) cast specimens.

Figure 4 shows the carbide precipitation behavior of the carburized region obtained in the cold-rolled and cast specimens. The microstructural behavior of each carburized specimen was investigated from the surface to the center area. For the cold-rolled specimen, the grain size (~7 μm) of the carburized specimen was coarser than that of the BM (~1 μm), as indicated in Figure 1a. Figure 4a indicates that fine carbides (Cr_7_C_3_) with green colour are densely distributed on the surface, which are periodically precipitated in specific regions toward the center area. From the enlarged images of the upper regions of the cold-rolled and carburized specimens, the carbides were evenly distributed regardless of the intragranular and grain boundaries. Additionally, they were mainly distributed along the grain boundaries as they approached the center region, as shown in austenitic stainless steel [23,24].

However, the carburization on the cast specimen with a coarse grain size produced a significant variation in the massed and lacked areas of fine carbides, regardless of the upper and lower regions of the specimen (Figure 4b). Coarse carbides are formed in the carbide-lacking area. A previous study reported that FCC materials have various carbon (and nitrogen) diffusion reactions depending on the crystal orientation, such as the (111) and (100) planes [25,26]. Comparing the two types of surfaces, because the activation energy of the dense (111) surface is larger than that of the more open (100) surface, it is relatively difficult to cause the reactions by carbon diffusion on the dense (111) surface [25]. Therefore, it was expected that the crystal orientations of the grains affected carbon diffusion, resulting in massed and lacked areas. In the cast specimen with coarse grains, however, the orientation of the adjacent grains was identical to the (100) surface, which is indicated by the pink colour in the IPF map (Figure 4b). In addition, the variation of the carbide formation in the cast specimen is under investigation and will be reported in a future study.

The carbide precipitation behavior of the cold-rolled specimen was similar to that of a previous study (carburization at 920 °C for 10 h) by Zhang et al. [13]. However, a solid solution-strengthened carburization layer was formed on the surface without carbides by carbon diffusion in Peng et al. [7], which was carburized at a low temperature (450 °C) for 40 h. Therefore, it is judged that the formation of carbides by carburization is more affected by the carburizing temperature than by the carburizing time.

### 3.3. Compositional Behavior of Carburized/Diffused Layer with Respect to the Grain Sizes of the BMs

**Figure 5:**
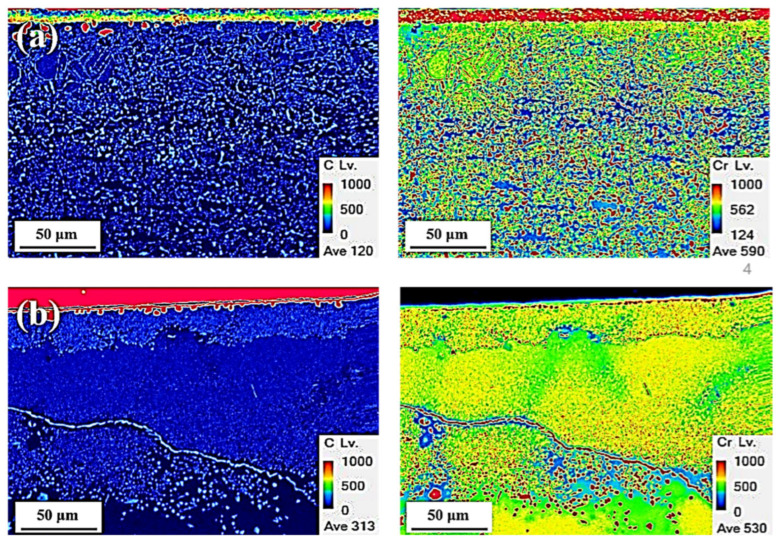
Compositional behavior of the carburized specimens: (**a**) cold-rolled and (**b**) cast specimens.

Figure 5 shows the macro-mapping results of the carburized layer obtained for the cold-rolled and cast specimens. The macro-mapping analyzed the same areas as those in Figure 2b,d. Figure 5a,b show the carburized specimens of the cold-rolled and cast HEAs, respectively. It was confirmed that each carburized specimen formed a carburized layer from the surface by carbon diffusion. According to the compositional behavior of the C and Cr components, the C-dense carburizing layers (10 μm of the cold-rolled and 5 μm of the cast specimen) in all specimens were approximately 40 times thinner than that expected from Figure 2 (400 μm of the cold-rolled and 200 μm of the cast specimen). The carburized layer with a dense C component and the diffused layer formed by diffusing the C component were clearly separated, and some carbon particles were present in all specimens. The C component seems to be mainly diffused through the grain boundaries, and the C component was evenly distributed inside the grain. In addition, the carburized layer and grain boundaries with dense C showed that the Cr component was abundant. Because the diffused layer of the cold-rolled HEA had very fine grains, the C and Cr components were evenly distributed around the grain boundaries, as shown in Figure 5a. However, the C and Cr components were mainly segregated along the grain boundaries in the diffused layer of the cast specimen with coarse grains, and the massed and lacking areas of the C and Cr components were present in the grains around the grain boundaries.

**Figure 6:**
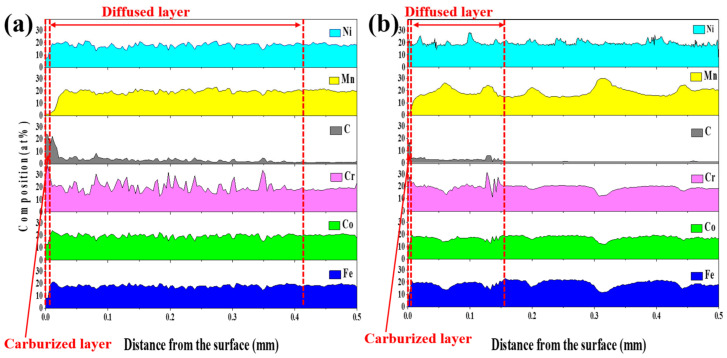
Line analysis of carburized layers from the surface: (**a**) Cold-rolled HEA and (**b**) cast HEA.

**Figure 7:**
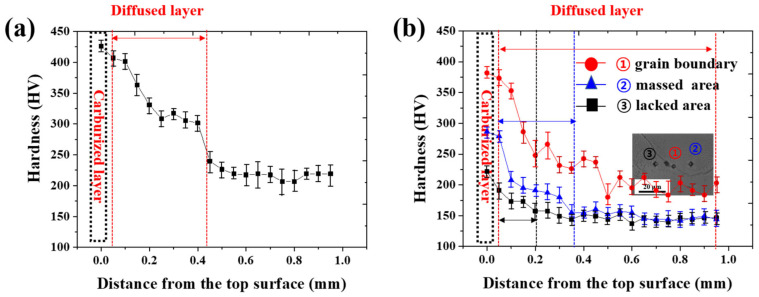
Hardness distribution of carburized specimens: (**a**) Cold-rolled and (**b**) cast HEA.

Figure 6 shows the compositional behavior of the carburized layer obtained for the cold-rolled and cast specimens. To analyze the compositional behavior between the carburized layer and the BM of all specimens, a line analysis was performed from the surface to the center area of the specimens. As shown in Figure 5, the carburized layer presented high contents of the C and Cr components in each specimen, and it was approximately 10 μm thick. Conversely, the carbon diffused layer of the cold-rolled specimen was formed approximately twice as deep as that of the cast specimen, as shown in Figure 2a,c. As mentioned above, the carburized and diffused layers in the carburized cast specimen were separated by the massed and lacked area of the carbides, so it was challenging to clearly determine the diffused depth of carbon. The high contents of C and Cr in the carburized and diffused layers of all specimens affected the contents of Ni, Mn, Co, and Fe. Especially in the carburized layer, the contents of C and Cr were significantly higher than those of the other components. These results indicate that carbon particles and Cr_7_C_3_ carbides are densely formed in the carburized layer. The carbon content decreased significantly in the diffused layer, but the carbon content did not disappear because the carbides were formed by the diffusion of carbon. As mentioned above, because the fastest diffusion of carbon occurred, most carbides were generated at the grain boundaries, and the results of the line analysis in the diffused layer showed an up/down cycle. In the diffused layer of the cold-rolled specimen, the contents of C and Cr increased in the grain boundaries, but the contents of Ni, Mn, Co, and Fe decreased, as shown in Figure 7a. Therefore, M_7_C_3_ and M_23_C_6_ observed in the XRD pattern results in Figure 3 are Cr_7_C_3_ and Cr_23_C_6_. Not only the contents of C and Cr but also the contents of Mn increased in the grain boundaries in the diffused layer of the cast specimen. However, the contents of Co and Fe showed opposite results. As the line analysis was performed in the absence of carbides, the effects of the C and Cr contents were less than those of the cold-rolled specimen. In addition, the grain sizes of the cast specimen were coarser than those of the cold-rolled specimen, so the width of the up/down cycle was larger than that of the cold-rolled specimen.

Figure 7 shows the hardness distribution of the carburized specimens obtained from the cold-rolled and cast HEAs. The average hardness of the cold-rolled HEA BM (approximately 324 HV_0.5_) was approximately 100 HV higher than that of the center area in the carburized specimen. The carburized layer on the upper surface of the cold-rolled specimen showed the highest hardness of approximately 430 HV_0.01_, and the hardness distribution tended to decrease toward the center area of the carburized specimen, as shown in Figure 7a. While approaching the matrix after carburization, the hardness distribution gradually decreased because the fraction of carbides decreased. From the upper surface of the carburized specimen to the region where the hardness becomes equivalent to that of the matrix, there are various areas affected by the diffusion of carbon. Because the massed and lacking areas of the carbides are divided from the grain boundaries, the hardness was measured from grain boundaries/massed/lacking areas, as shown in Figure 7b. The maximum hardness (approximately 382/286/222 HV_0.01_) was measured at the grain boundaries, massed, and lacking areas, respectively, and the hardness gradually decreased toward the matrix (about 200/146/144 HV_0.01_). Because the grain boundaries are the main diffusion path of the carbon, high-density carbides of the C and Cr contents were formed, and it was confirmed that the hardness along the grain boundary was higher than that of the massed and lacking areas. The width of the carbon diffused layer tends to increase with the width of the hardness distribution in the grain boundaries/massed/lacking areas. A diffused layer of approximately 200 μm was formed in the carbide-lacking areas, as shown in Figure 5b. However, in the carbide-massed area, the carbon diffused to a depth of approximately 400 μm to form carbides, which increased the range of the diffused layer, as shown in Figure 7b. A diffused layer of approximately 1 mm was formed at the grain boundaries. In addition, the hardness distribution of the grain boundaries on the carburized cast specimen showed a tendency similar to that of the carburized cold-rolled specimen. The grain boundaries offer the easiest diffusion paths for carbon; therefore, carbides are formed along the grain boundaries from the upper surface to the center area, and the hardness distribution along the grain boundary is measured to be higher than that of the matrix.

## 4. Conclusions

In this study, the carburization characteristics of cold-rolled and cast high-entropy alloys with various grain sizes are investigated. The main results are as follows.

(1) The carburization region formed in the carburized cold-rolled specimen was deeper than that of the cast specimen, and the carbides of the carburized/diffused layer mainly comprised Cr_7_C_3_ regardless of the BM type.
(2) The carburized layers of all specimens were formed as thin as 10 μm, and the carburized layer was clearly divided from the diffused layer. The C content was mainly diffused along the grain boundaries, and the C content was evenly distributed inside the grain for the cold-rolled specimen. However, in the carburized cast specimen, the C content was unequally distributed to the massed grain that diffused from the grain boundaries.
(3) The surfaces of all carburized specimen were composed of a carburized layer with high C and Cr contents; therefore, the Cr_7_C_3_ carbides were distributed evenly regardless of the grain boundary and interior. Furthermore, in the diffused layer, the carbides were mainly formed along the grain boundaries for the cold-rolled specimen and the carbide regions were divided into the grain boundaries, grain boundaries, massed, and lacking areas, for the cast specimen.
(4) Owing to the non-uniform formation of carbides in the carburized cast specimen, the areas in the diffused layer exhibited various carbide densities and hardness distributions. Therefore, to improve the carburization efficiency of equiatomic CoCrFeMnNi high-entropy alloys, it is necessary to refine the grain sizes.

## Data Availability

The data are available from the author upon request.
